# Anti-PD-1 immunotherapy leads to tuberculosis reactivation via dysregulation of TNF-α

**DOI:** 10.7554/eLife.52668

**Published:** 2020-02-24

**Authors:** Liku B Tezera, Magdalena K Bielecka, Paul Ogongo, Naomi F Walker, Matthew Ellis, Diana J Garay-Baquero, Kristian Thomas, Michaela T Reichmann, David A Johnston, Katalin Andrea Wilkinson, Mohamed Ahmed, Sanjay Jogai, Suwan N Jayasinghe, Robert J Wilkinson, Salah Mansour, Gareth J Thomas, Christian H Ottensmeier, Alasdair Leslie, Paul T Elkington

**Affiliations:** 1 NIHR Biomedical Research Centre, School of Clinical and Experimental Sciences, Faculty of Medicine, University of SouthamptonSouthamptonUnited Kingdom; 2 Institute for Life Sciences, University of SouthamptonSouthamptonUnited Kingdom; 3 Africa Health Research InstituteKwaZulu NatalSouth Africa; 4 Department of Tropical and Infectious Diseases, Institute of Primate Research, National Museums of KenyaNairobiKenya; 5 Wellcome Centre for Infectious Diseases Research in Africa, Institute of Infectious Disease and Molecular Medicine, University of Cape TownCape TownSouth Africa; 6 TB Centre and Department of Clinical Research, London School of Hygiene and Tropical MedicineLondonUnited Kingdom; 7 Department of Clinical Sciences, Liverpool School of Tropical MedicineLiverpoolUnited Kingdom; 8 NIHR Biomedical Research Centre, School of Cancer Sciences, University of SouthamptonSouthamptonUnited Kingdom; 9 The Francis Crick InstituteLondonUnited Kingdom; 10 BioPhysics Group, Department of Mechanical Engineering, University College LondonLondonUnited Kingdom; 11 Department of Infectious Diseases, Imperial College LondonLondonUnited Kingdom; 12 Department of Infection and Immunity, University College LondonLondonUnited Kingdom; Tufts University School of MedicineUnited States; Institute of Industrial Science, The University of TokyoJapan

**Keywords:** tuberculosis, pathology, immune checkpoint inhibitors, TNF, host-pathogen interaction, Human

## Abstract

Previously, we developed a 3-dimensional cell culture model of human tuberculosis (TB) and demonstrated its potential to interrogate the host-pathogen interaction (Tezera et al., 2017a). Here, we use the model to investigate mechanisms whereby immune checkpoint therapy for cancer paradoxically activates TB infection. In patients, PD-1 is expressed in *Mycobacterium tuberculosis* (Mtb)-infected lung tissue but is absent in areas of immunopathology. In the microsphere model, PD-1 ligands are up-regulated by infection, and the PD-1/PD-L1 axis is further induced by hypoxia. Inhibition of PD-1 signalling increases Mtb growth, and augments cytokine secretion. TNF-α is responsible for accelerated Mtb growth, and TNF-α neutralisation reverses augmented Mtb growth caused by anti-PD-1 treatment. In human TB, pulmonary TNF-α immunoreactivity is increased and circulating PD-1 expression negatively correlates with sputum TNF-α concentrations. Together, our findings demonstrate that PD-1 regulates the immune response in TB, and inhibition of PD-1 accelerates Mtb growth via excessive TNF-α secretion.

## Introduction

Tuberculosis (TB) continues to be a global health pandemic, killing more people than any other infection (Wallis et al., 2016). TB involves a complex host-pathogen interaction, with humans and *Mycobacterium tuberculosis* (Mtb) having undergone prolonged co-evolution (Menardo et al., 2019). TB has often been thought to primarily result from loss of immune control, because approximately 90% individuals infected with TB never progress to active disease, and this progression is increased in the context of immune deficiency; such as in cases of HIV infection, in infants, people with genetic deficiency of the IL-12/IFN-γ signalling pathway or after anti-TNF-α antibody treatment (O'Garra et al., 2013). However, an emerging concept is that an excessive immune response to Mtb may be equally harmful. Standard disease paradigms predict that immune activation resulting from the administration of checkpoint inhibitors should lead to better control of Mtb infection (Zumla et al., 2016). However, counter-intuitively these agents seem to be activating TB, as evidenced by recent reports of TB developing in patients treated for malignancy with immune checkpoint inhibition, often rapidly after commencing therapy (Fujita et al., 2016; Lee et al., 2016; Chu et al., 2017; Picchi et al., 2018; Jensen et al., 2018; Elkington et al., 2018; He et al., 2018; Takata et al., 2019; Barber et al., 2019; Tsai et al., 2019; van Eeden et al., 2019). Consistent with this emerging clinical phenomenon, programmed death (PD-1) deficient mice are highly susceptible to TB, dying more rapidly than T-cell deficient mice (Lázár-Molnár et al., 2010; Barber et al., 2011).

PD-1 and its ligand PD-L1 are expressed in human granulomas (Elkington et al., 2018), suggesting a regulatory role at the site of disease. TB granulomas are hypoxic (Belton et al., 2016), and PD-L1 is up-regulated by hypoxia (Noman et al., 2014), further suggesting a mechanistic link between hypoxia and the PD-1/PD-L1 axis within TB lesions. In this study, we investigate the expression patterns of PD-1 and PDL-1 within TB infected human lung tissue and the relationship between PD-1 and anti-TB immunity. Next, using a human 3D cell culture model of TB (Tezera et al., 2017a), we show that hypoxia increases expression of PD-1 and its ligands, that PD-1 inhibition increases Mtb growth. Surprisingly, TNF-α is primarily responsible for this effect, and TNF-α neutralisation reverses the anti-PD-1 induced phenotype.

## Results

### PD-1 is expressed in human TB granulomas but not in areas of immunopathology

**Figure 1.**
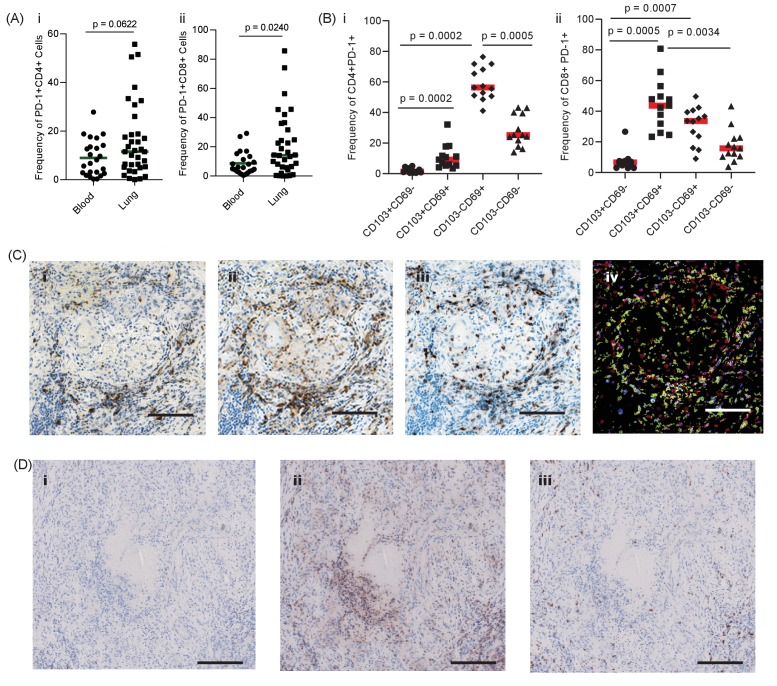
PD-1 is expressed in human TB granulomas. (**A**) Analysis of PD-1 expression by T cells in the lung and peripheral circulation of thirty-five TB patients undergoing medically indicated lung resection. PD-1 shows a trend towards higher expression by lung CD4^+^ (**i**) and is significantly higher on lung CD8^+^ (ii) T cells. Significance analysed by one-tailed unpaired Mann-Whitney test. (**B**) Flow cytometric analysis of lung parenchyma CD4^+^ (i) and CD8^+^ (ii) T cells from TB patients based on the expression of PD-1, CD69 and CD103 demonstrates increased PD-1 expression in the resident T cells in the lung parenchymal cells. Significance analysed by Kruskal-Wallis test with Dunn’s multiple comparison test. (**C**) Immunohistochemical staining for PD-1^+^, CD4^+^ and CD8^+^ expression in human lung TB granulomas. PD-1 is expressed around the central macrophage core in the same region as CD4^+^ (ii) and CD8^+^ (iii) T cells. Co-localization of PD-1 (blue), CD4^+^ (red) and CD8^+^ (yellow) using false colour of the immunostaining shows co-localisation of PD-1 with both CD4^+^ and CD8^+^ cells (purple and green respectively) (iv). Scale bar 100 μm. (**D**) PD-1 is not expressed in caseating granulomas where immunopathology is present in human lung biopsies (i). Six biopsies taken as part of routine clinical care were studied. CD4^+^ (ii) and CD8^+^ (iii) expressing cells are present in the same area, and so absence of PD-1 immunoreactivity is not due to lack of viable cells. Scale bar 200 μm.

First, we investigated the presence and localisation of PD-1-expressing T cells in human pulmonary TB. We hypothesised that PD-1 would be expressed by T cells in the lung of patients with TB, and at a higher frequency than in the blood. In thirty-five patients undergoing medically indicated lung resection to treat TB or TB sequalae, PD-1 expression was measured on T cells isolated from the lung and matched blood samples, available for 23 patients, by flow cytometry. Overall, PD-1 expression in homogenized lung tissue was highly variable, with a trend towards increased PD-1 in both CD4 and CD8 T-cells from the lung compared to matched blood, which reached statistical significance for CD8 T-cells (Figure 1A). Lung tissue from healthy individuals was not available for study, however, the median frequency of 11% and 14% of PD-1+ CD4 and CD8 T-cells observed are generally lower than recently reported for healthy human lung tissue from organ donors of approximately 50% for both cell types (Snyder et al., 2019). As lung tissue is highly perfused with blood, distinguishing cells of lung or blood origin is challenging.

**Figure 1—figure supplement 1.**
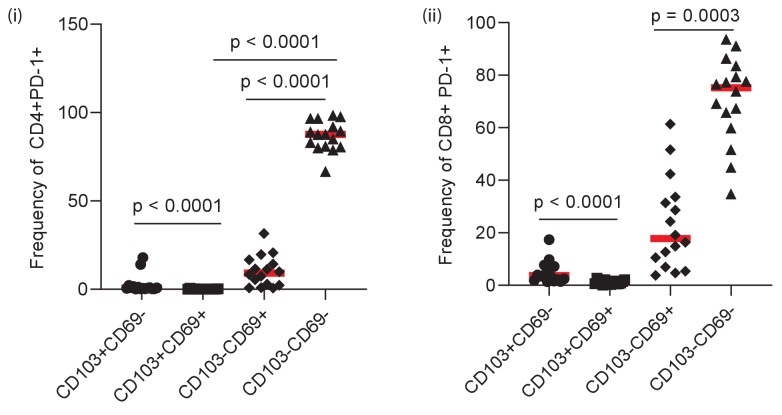
PD-1 expression on peripheral CD4^+^ and CD8^+^ T-cells is predominantly on CD103- and CD69-negative cells. Significance was analyzed by one-tailed unpaired Mann-Whitney test.

**Figure 1—figure supplement 2.**
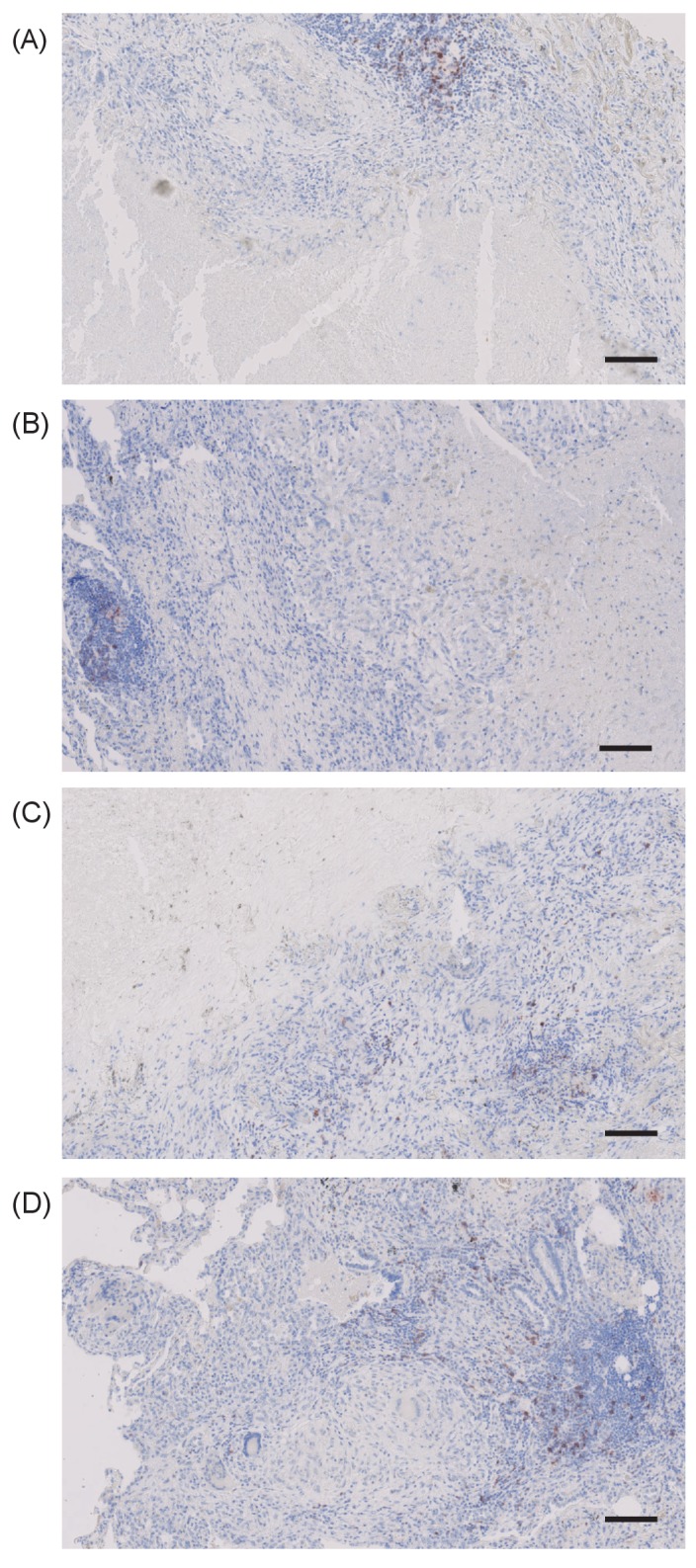
PD-1 expressing cells are absent in the immediate region surrounding caseous necrosis in human TB granulomas. Images from four different clinical samples are presented. Scale bar 100 μm.

To explore this further, we stained lung samples for canonical markers of tissue resident T-cells, CD69 and CD103 (Snyder et al., 2019). PD-1 expression on lung CD4 cells was predominantly restricted CD69+ T-cells, with a smaller proportion also expressing CD103 (Figure 1B, overall frequencies Supplementary file 1), consistent with a tissue resident phenotype and in contrast to PD-1 expressing cells in blood, which are largely CD69 negative (Figure 1—figure supplement 1). Lung CD8 T-cells were also found to predominantly express CD69 in addition to CD103, again in contrast to CD8 T-cells in circulation. Therefore, these data are consistent with expression of PD-1 on lung tissue resident T-cells in patients with active or previous pulmonary TB infection. However, TB immunopathology is highly heterogenous within the lung and these analyses do not provide information as to the localization of PD-1 expression within TB lesions.

To address this question, we performed immunohistochemical analysis of human lung biopsies of patients with active TB. Within stable granulomas with an intact cellular structure, PD-1 expression was present around the central macrophage core (Figure 1C–i). Both CD4 and CD8 T cells were present in the granuloma (Figure 1C–ii and -iii), and co-localisation analysis demonstrated that a proportion of both T cell subtypes expressed PD-1 (Figure 1C-iv), consistent with the flow cytometry data. In contrast, in caseating granulomas with evident immunopathology, PD-1 expression was totally absent (Figure 1D–i and Figure 1—figure supplement 2). The lack of immunoreactivity was not due to lack of viable cells, as CD4 and CD8 expressing T cells were present within the same area (Figure 1D–ii and -iii). Therefore, in human granulomas, PD-1 is expressed in areas where the host-pathogen interaction appears stable but is absent in regions of immunopathology.

### The PD-1/PD-L1 axis is up-regulated in the 3D microsphere model

**Figure 2.**
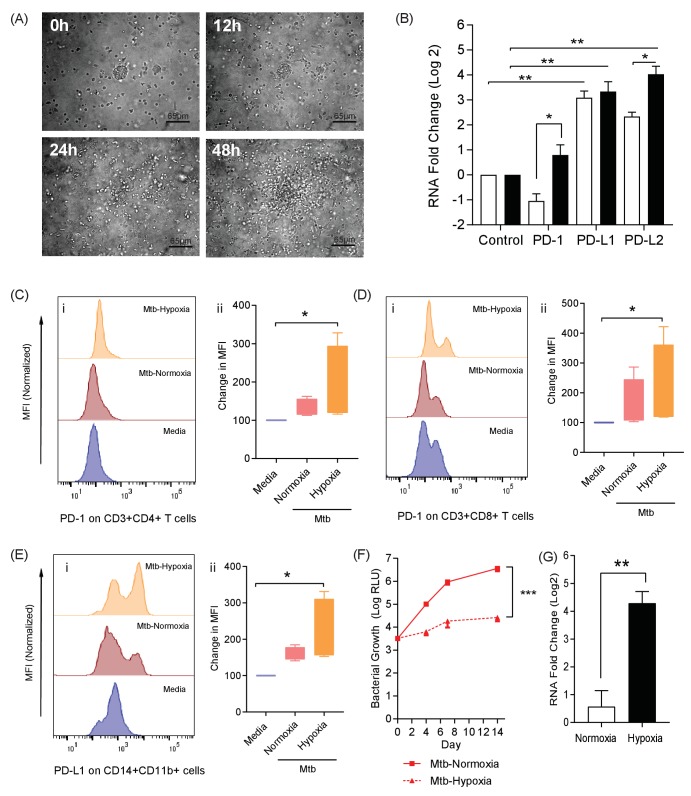
The PD-1/PD-L1 axis is upregulated in the 3D TB granuloma system. (**A**) Still images from time-lapse microscopy imaging demonstrating increasing cellular aggregation of PBMC around a focus of ultraviolet killed Mtb H37Rv in the 3D granuloma system at times 0, 12, 24 and 48 hr post encapsulation in the matrix. The Z projection shows the cells contained within the designated volume in a 2D reconstruction. Full time course in Video 1. (**B**) Gene expression of PD-1 and its ligands in the 3D microsphere model. RNA was extracted from live Mtb-infected PBMC and relative expression investigated by qRT-PCR at day four post infection. Open bars, normoxia, filled bars 1% hypoxia. PD-L1 and PD-L2 are upregulated by Mtb infection, and in 1% hypoxia PD-1 expression is increased and PD-L2 expression further augmented (n = 4). Results are normalised against the housekeeping genes GAPDH, β-Microbulin and FANTA and showed similar results. β-microglobulin used for (**B**). *p<0.05, **p<0.01. (**C–E**) Surface expression of PD-1 and PD-L1. PBMCs were decapsulated from Mtb-infected microspheres at day seven and surface expression of PD-1 and its ligand PD-L1 were analysed by flow cytometry. PD-1 is expressed in CD4^+^ (**C**) and CD8^+^(**D**) T cells in PBMC from Mtb infected microspheres incubated in normoxia. PD-1 expression was significantly upregulated in 1% hypoxia. Representative flow cytometry plots and level of expression of PD-1 by the CD4^+^ and CD8^+^ T cell fractions are shown (n = 4). (**E**) PD-L1 expression on CD14^+^CD11b^+^ cells within PBMC in Mtb infected microspheres is upregulated in both normoxia and 1% hypoxia at day 7 (n = 4). Significance of *p<0.05. (**F**) Growth of Mtb H37Rv ffLux+ in microspheres in normoxia and 1% hypoxia measured at day 3, 7 and 14. Hypoxia reduces Mtb growth. (**G**) Hypoxia inducible factor 1α (HIF-1α) mRNA levels were increased in Mtb-infected microspheres incubated in 1% hypoxia. RNA was extracted from decapsulated microspheres and normalised to uninfected microspheres in the same environment. Results were normalised to the housekeeping genes GAPDH, β-microglobulin and FANTA to check the housekeeping gene are not affected by hypoxia. Similar results all three of the housekeeping genes. β-microglobulin used for this graph. Significance ***p<0.001.

Taken together, these data from Mtb infected patients demonstrate that PD-1 is expressed by lung resident T-cells and may be required to prevent destructive lung disease. However, whether this is a causal associated remains unclear. We therefore explored the biological role of PD-1 expression in a 3-dimensional (3D) human tissue culture model. TB is a human disease characterised by a prolonged host-pathogen interaction in 3D, and is regulated by the extracellular matrix, and previously we have previously developed a 3D cell culture model incorporating extracellular matrix to investigate TB pathogenesis (Tezera et al., 2017a; Tezera et al., 2017b). Here, we first studied migration of cells within the alginate-collagen matrix by performing time-lapse microscopy (Figure 2A and Video 1). For this experiment only, UV killed Mtb was used to permit the imaging outside the containment level three laboratory. From 24 hr, progressive accumulation occurs around a central infected core, resulting in a large multicellular granuloma by 48 hr with dynamic cellular movement similar to the T cell mobility observed in mouse BCG granulomas (Egen et al., 2008).

**Figure 2—figure supplement 1.**
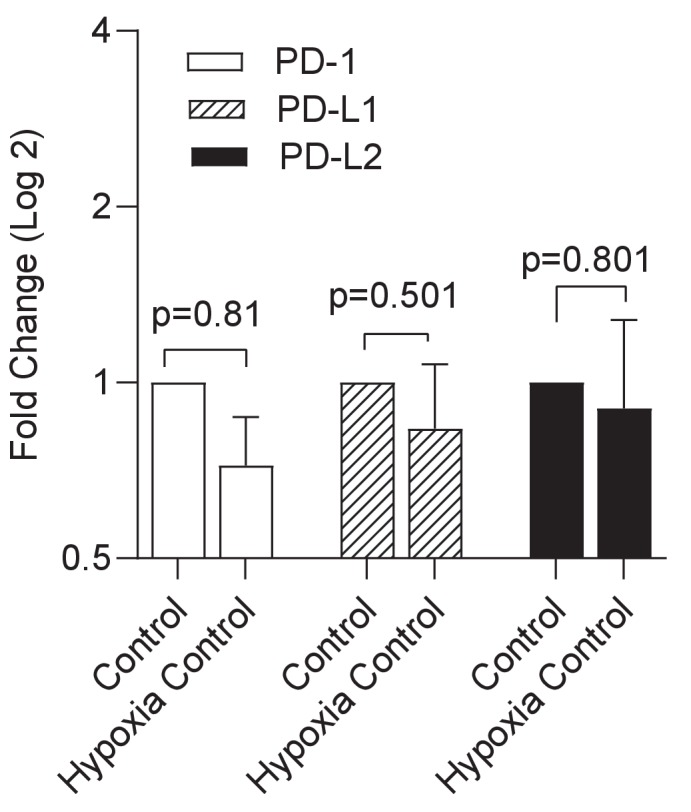
Hypoxia alone has no significant effect on expression of the PD-1/PD-L1/2 axis. Gene expression PD-1 and its ligands in uninfected cells was compared between normoxic and hypoxic conditions in the microsphere model. RNA was extracted from uninfected PBMCs and relative expression investigated by qRT-PCR at day 4. Open bars PD-1, filled bars PD-L1 and black bars PD-L2 respectively. Results were normalised against the housekeeping genes GAPDH, β-Microbulin and FANTA and showed similar results. No significant changes in gene expression were noted in uninfected microspheres. β-microglobulin used for the analysis presented.

**Figure 2—figure supplement 2.**
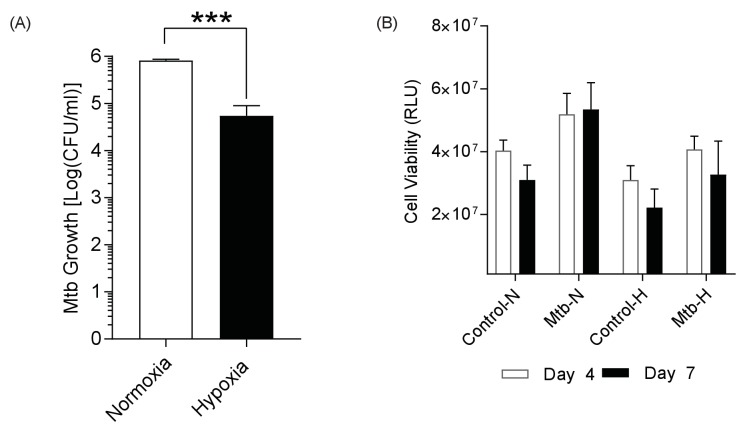
Mtb growth is reduced in hypoxia, while host cell viability is unchanged. (**A**) Mtb H37Rv ffluc+ growth in 1% hypoxia. Microspheres were decapsulated in a media containing 5 mM EDTA and 0.1% saponin at day 14. The lysate was centrifuged, re-suspended in 1 ml PBS, serially diluted and grown in Middlebrook 7H11 media for 3 weeks before colony counting. (**B**) Host cell viability using 3D CytoxGlo viability assay (Promega) shows no significant difference between normoxia (open bars) and hypoxia (filled bars) at day 4 and day 7.

**Video 1.** Cell migration over 48 hr around a central cluster of macrophages infected with UV-killed Mtb within a 3D alginate-collagen matrix. Migration is seen in the first 24 hr, without aggregation, and then progressive granuloma formation occurs.

We next investigated whether the PD-1/PD-L1 axis was up-regulated by infection in this 3D model, and found that Mtb infection increased PD-L1 and PD-L2 gene expression at 4 days post infection (Figure 2B). Furthermore, a hypoxic environment (1% oxygen) further increased PD-L2 expression. PD-1 gene expression was not increased at 72 hr by Mtb infection under normoxic conditions, but was up-regulated when infected cells were incubated in hypoxia (Figure 2B). The change in expression required both infection and hypoxia, as hypoxia did not increase PD-1 or PD-L1/2 expression alone (Figure 2—figure supplement 1). We then studied PD-1/PD-L1 cellular surface expression in microspheres by flow cytometry after one week of infection. Mtb infection slightly increased PD-1 surface expression on CD4^+^ T cells in normoxia, which was augmented by hypoxia (Figure 2C). Similarly, Mtb infection increased PD-1 expression on CD8^+^ T cells, which was greater in hypoxia (Figure 2D). Furthermore, on CD14^+^ CD11b^+^ cells, PD-L1 expression was increased by infection and more so in hypoxia (Figure 2E). To investigate whether the effect of hypoxia on increased PD-1/PD-L1 expression was due to altered Mtb growth, we studied proliferation using Mtb expressing firefly luciferase and also colony counting on Middlebrook 7H11 agar. Hypoxia inhibited Mtb growth by both readouts (Figure 2F and Figure 2—figure supplement 2A). As expected, hypoxia also increased expression of the hypoxia inducible factor, HIF-1α, in host cells (Figure 2G), but did not have a significant effect on host cell survival (Figure 2—figure supplement 2B). Therefore, the increased PD-1 and PD-L1/2 expression in hypoxia was not due to increased Mtb growth or a change in host cell viability.

### PD-1 pathway inhibition increases Mtb growth in the 3D model

**Figure 3.**
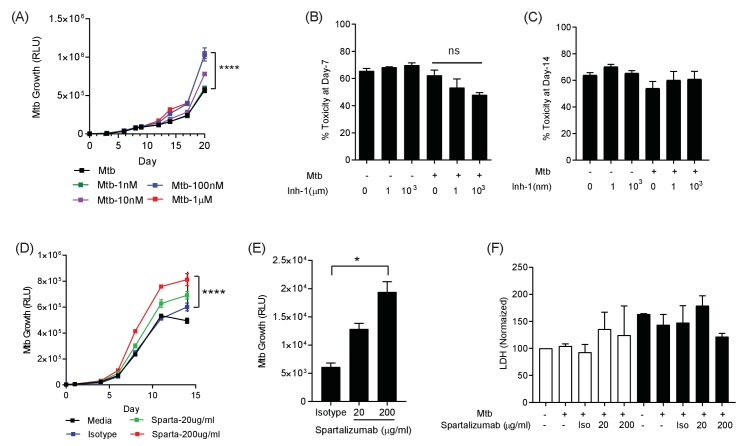
PD-1 pathway inhibition increases Mtb growth. (**A**) Inhibition of PD-1 receptors by small chemical inhibitor one increases Mtb growth in a dose-response manner (1–1000 nM). Inhibitor concentration 1 nM (green), 10 nM (purple), 100 nM (blue) and 1 µM (red). (**B**) Inhibitor one was not toxic to Mtb-infected PBMC, analysed by CytoTox-Glo assay (Day 7). (**C**) Cellular toxicity was no different at day 14 as analysed by LDH release. Concentration 1 and 1000 nM were analysed for toxicity. (**D**) Spartalizumab, a therapeutic monoclonal anti-PD-1 antibody, progressively increased Mtb growth in microspheres in normoxia in a dose-dependent manner. (**E**) Spartalizumab also increased Mtb growth in hypoxia. Media (black), isotype (blue), spartalizumab 20 µg/ml (green) and 200 µg/ml (red). (**F**) The anti-PD-1 antibody had no effect on cell survival in microspheres in normoxia (clear bars) and 1% hypoxia (filled bars). Cytotoxity is determined by measuring LDH release at day 14 and normalized by the control. ****p<0.0001.

Having demonstrated that the PD-1/PD-L1 axis was up-regulated by infection in the 3D model, we investigated whether inhibition of this interaction modulated host control of Mtb. We initially studied chemical inhibitors of the PD-1/PD-L1 axis (Inhibitor 1, C_29_H_33_NO_5_) and found that blocking PD-1/PD-L1 binding increased Mtb growth in a dose-dependent manner (Figure 3A). This effect occurred in both normoxia and hypoxia (Figure 3—figure supplement 1). No effect of PD-1 inhibition on cell survival was noted at day 7 or day 14, utilising two different assays suited to each time point (Figure 3B and C). Next, we studied spartalizumab, a humanised monoclonal antibody with high-affinity to PD-1 that blocks the interaction with PD-L1 and PD-L2 (Kaplon and Reichert, 2019). Consistent with the chemical inhibition, PD-1 pathway inhibition using spartalizumab increased Mtb growth in normoxia in a dose-dependent manner (Figure 3D). Similarly, in hypoxia spartalizumab increased Mtb growth (Figure 3E). Microspheres must be restored to normoxia to measure Mtb luminescence, and therefore a single time point was analysed. Anti-PD-1 antibody treatment had no significant effect on cell survival in either normoxia or hypoxia (Figure 3F).

**Figure 3—figure supplement 1.**
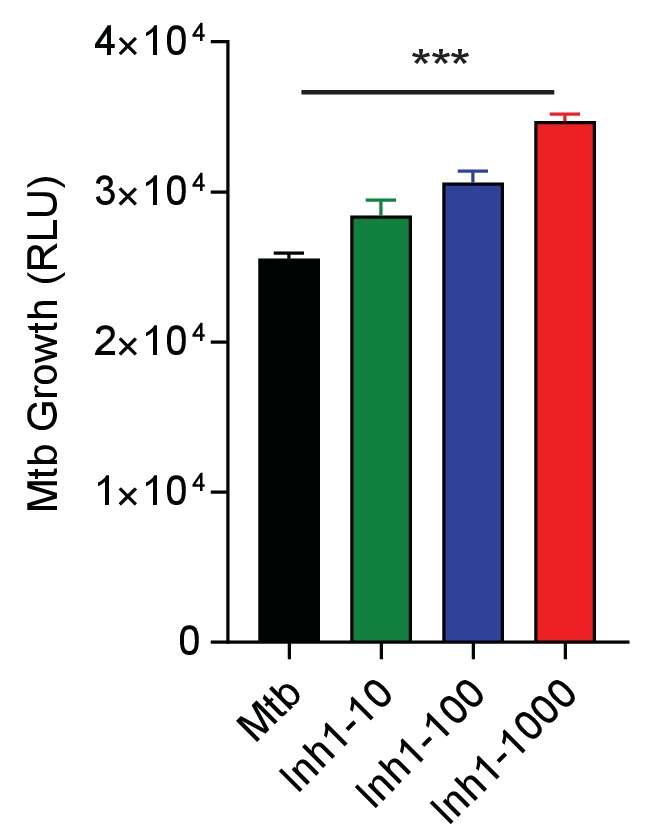
Small chemical inhibition of PD-1/PD-L1 interaction in 1% hypoxia measured at day 14 shows a dose-dependent increase in Mtb growth with PD-1 inhibition. ***p<0.0001 with one-way ANOVA with Dunnett’s multiple comparison test.

### PD-1 inhibition increases secretion of multiple cytokines and growth factors

**Figure 4.**
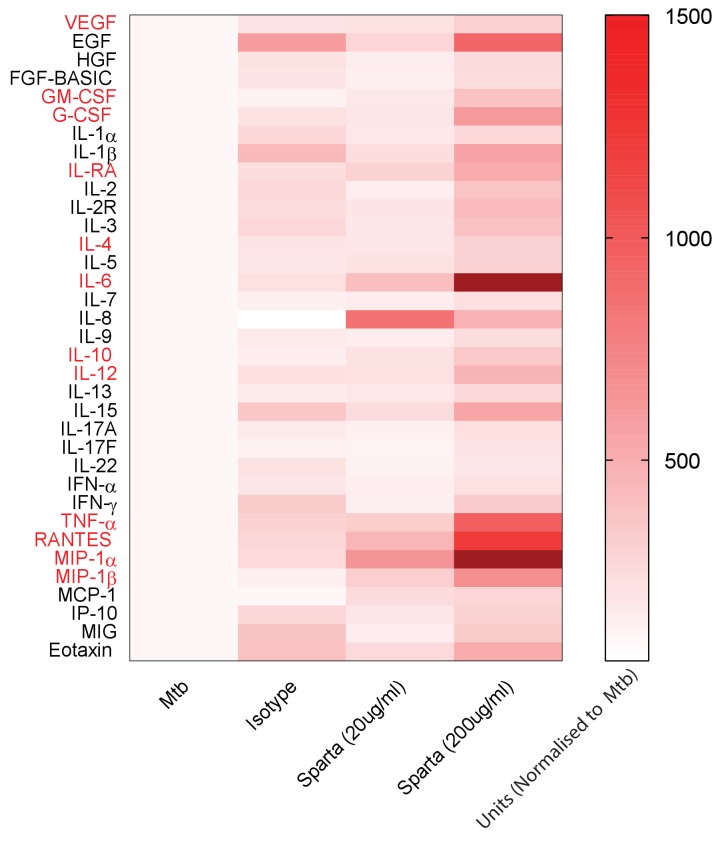
PD-1 inhibition increases secretion of multiple cytokines and growth factors. PD-1/PD-L1 signalling was inhibited by Spartalizumab, a humanized IgG4 anti-PD1 monoclonal antibody, in Mtb-infected microspheres at 20 and 200µ/ml in normoxia. Supernatants were collected at day 14 and accumulation of cytokines and growth factors was analysed by Luminex 35-multiplex assay. Concentrations were normalized to secretion by Mtb infected microspheres to demonstrate relative fold change, and individual concentrations are shown in Figure 4—figure supplement 1. The experiment was performed twice with three replicates. Red font: **p<0.001 for Spartalizumab versus isotype control.

To investigate the underlying mechanism whereby PD-1 inhibition leads to increased Mtb growth, we studied secretion of cytokines, chemokines and growth factors by measuring accumulation in the media around Mtb-infected microspheres by Luminex array. Mtb infection increased secretion of numerous analytes (Figure 4—figure supplement 1), and inhibition of PD-1/PD-L1 signalling further significantly augmented secretion of twelve analytes compared to Mtb infection alone (Figure 4). A similar augmentation of analyte secretion was observed in hypoxic microspheres (Figure 4—figure supplement 2). The twelve analytes that were further increased above Mtb infection with concurrent PD-1 inhibition were IL-4, IL-6, IL-10, IL-12, TNF-α, IL-1RA, MIP-1α, MIP-1β, RANTES, G-CSF, GM-CSF and VEGF (individual concentrations, Figure 4—figure supplement 1). Anti-PD-1 alone increased TNF-α secretion, although to a lesser effect than in combination with Mtb infection (Figure 4—figure supplement 3).

**Figure 4—figure supplement 1.**
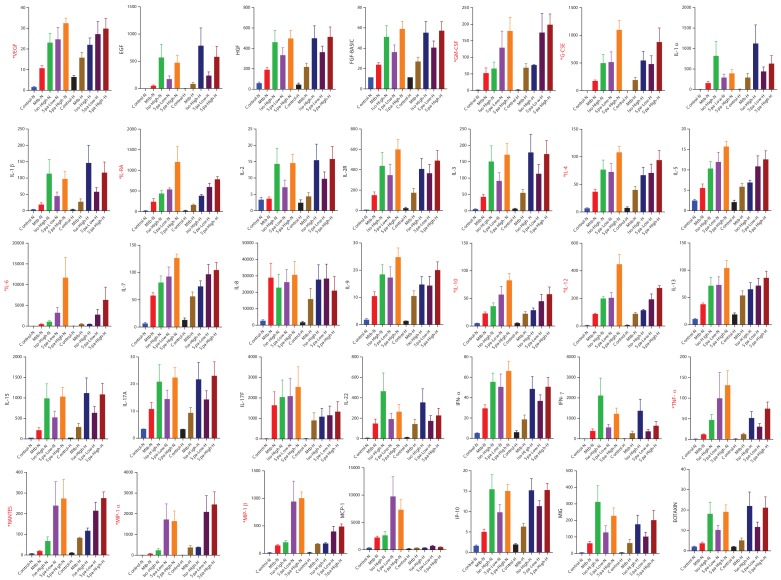
Cytokine accumulation around microspheres after inhibition of PD-1/PD-L1 signalling with Spartalizumab, a humanized IgG4 anti-PD1 monoclonal antibody at 20 and 200 µg/ml in normoxia (N) and 1% hypoxia (H). Supernatants were collected at day 14 and a Luminex 35-multiplex assay was performed. The experiment was performed twice with three replicates. Concentrations are in pg/ml. Labels in red correspond to significantly raised cytokine values. Red font: Significance **p<0.001.

**Figure 4—figure supplement 2.**
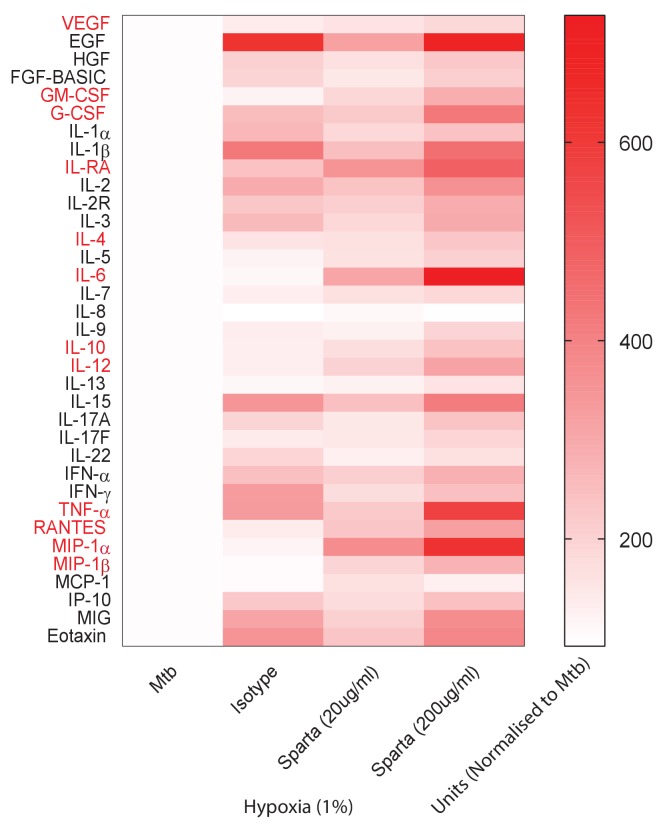
PD-1 inhibition increases secretion of multiple cytokines and growth factors in 1% hypoxia. Supernatants were collected at day 14 and accumulation of cytokines and growth factors was analysed by Luminex 35-multiplex assay. Concentrations were normalized to secretion by Mtb infected microspheres to demonstrate relative fold change, and individual concentrations are shown in Figure 4—figure supplement 1. The experiment was performed twice with three replicates. Red font: **p<0.001 for Spartalizumab versus Isotype control.

**Figure 4—figure supplement 3.**
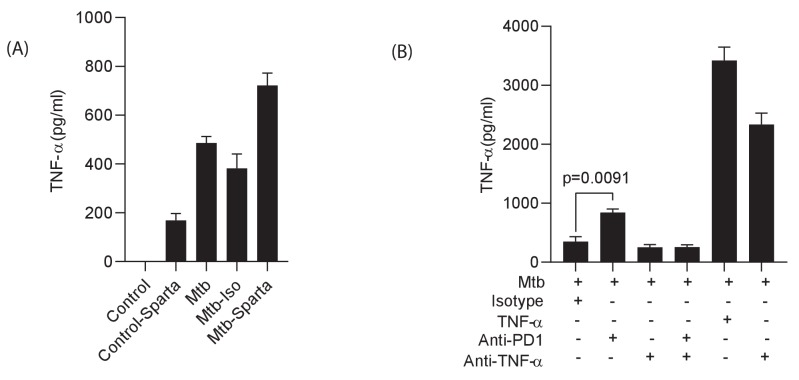
Spartalizumab induces TNF-α secretion in uninfected and infected microspheres, which is neutralised by anti-TNF-α. PBMCs which were uninfected or infected with Mtb were encapsulated in alginate-collagen matrix after pre- incubation in Spartalizumab at 200 μg/ml. The supernatant was collected to measure TNF-α secretion at day 7. (**A**) Spartalizumab induces TNF-α secretion in uninfected cells above background, which is accentuated with Mtb infection. (**B**) Anti-PD-1 antibody increases TNF-α secretion from Mtb infected cells above isotype, and the detectable levels are suppressed by anti-TNF antibody (50 μg/ml).

### Exogenous TNF-α increases Mtb growth in microspheres

**Figure 5.**
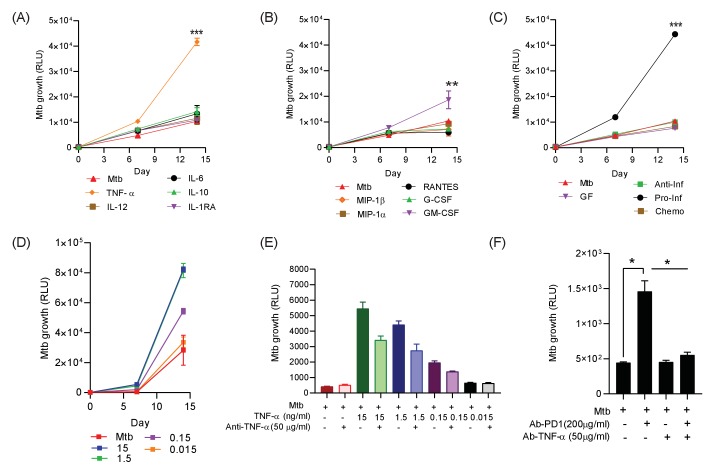
Addition of TNF-α increases Mtb growth in microspheres in normoxia. Recombinant human G-CSF, GM-CSF, IL-4, IL-6, IL-10, IL-12, TNF-α, IL-1RA, MIP-1α, MIP-1β or RANTES were added either individually (**A** **and B**) or in combination pools (**C**) to Mtb-infected microspheres at ‘low’ concentrations, defined as that measured in media around spheres after anti-PD-1 treatment. Recombinant human TNF-α increases growth of Mtb, whilst other pro-inflammatory cytokines did not (**A**). GM-CSF has a lesser growth-promoting effect (**B**). The only combination pool that increased Mtb growth was the pro-inflammatory cytokine pool, containing TNF-α (**C**). (**D**) TNF-α results in a dose-dependent increase in the Mtb growth over time. (**E**) Anti-TNF-α neutralising antibodies partially suppress the increased Mtb growth caused by TNF-α augmentation. Anti-TNF-α from Thermo Fisher Scientific. (**F**) Anti-PD1 antibody incorporation within microspheres increases of Mtb growth at day 7, and this effect is reversed by concurrent anti-TNF-α neutralising antibodies within microspheres. The constituent of the cytokine pools are: Growth factor pool (GF: GM-CSF and G-CSF), Anti-Inflammatory cytokine pool (Anti-Inf: IL-10 and IL-1RA), Pro-Inflammatory cytokine pool (Pro-Inf: TNF-α, IL-6 and IL-12) and Chemokine pool (Chemo: RANTES, MIP-1α, MIP-1β).

To establish which of these factors might be associated with increased Mtb growth, we added the significantly upregulated analytes either individually or in combination pools to 3D microspheres in normoxia at ‘low’ (Figure 5) and ‘high’ concentrations (Figure 5—figure supplement 1), as determined by the concentration measured in the secretion analysis. TNF-α was the dominant cytokine that increased Mtb growth, with other cytokines only having a minor effect (Figure 5A). Chemokines (RANTES, MIP-1α and MIP-1β) and growth factors (G-CSF) had no significant effect, while GM-CSF significantly increasing Mtb growth, but the effect was smaller than for TNF-α (Figure 5B). Additionally, the only cytokine combination that had a significant effect on Mtb growth was the pro-inflammatory pool containing TNF-α, IL-6 and IL-12 (Figure 5C). The addition of Th_2_ cytokines, chemokines or other growth factors had no significant effect (individual growth curves at ‘high’ concentration, Figure 5—figure supplement 1). Furthermore, TNF-α had a progressive dose-dependent effect increasing Mtb growth within microspheres (Figure 5D).

**Figure 5—figure supplement 1.**
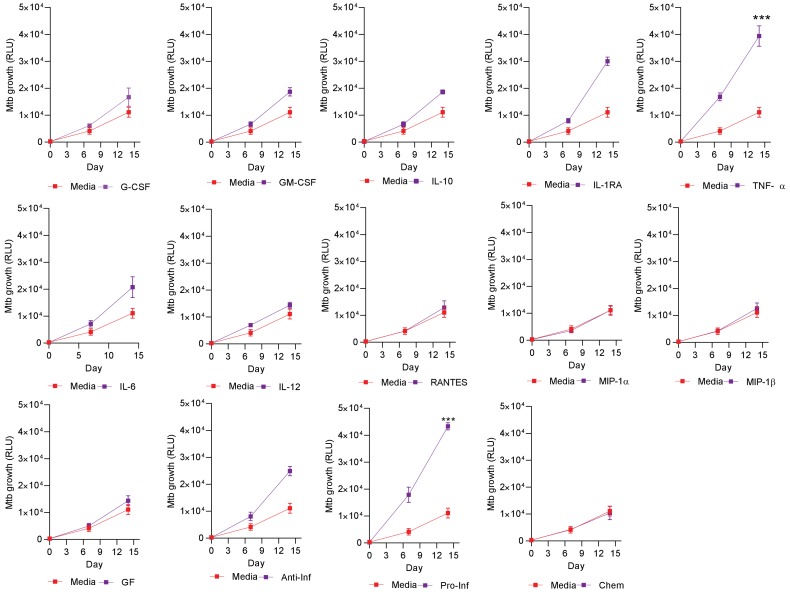
Individual Mtb growth curves at ‘high’ cytokine concentration, five times the concentration measured in media after anti-PD-1 treatment. Human recombinant G-CSF, GM-CSF, IL-4, IL-6, IL-10, IL-12, TNF-α, IL-1RA, MIP-1α, MIP-1β and RANTES were added to microspheres either individually or in combination pools to microspheres at five times the concentrations in Figure 5, determined by the concentration measured in the media around the microspheres. TNF-α increases Mtb growth in microspheres alone and in the pro-inflammatory pool.

**Figure 5—figure supplement 2.**
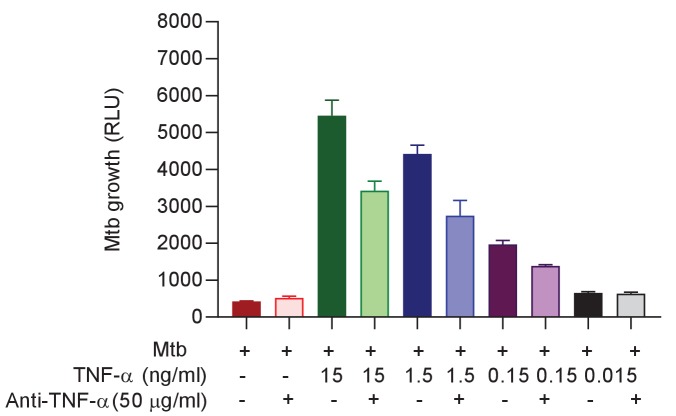
Anti-TNF-α neutralizing antibodies supress the Mtb growth following TNF-α from a different source (Anti-TNF-α from Sigma-Aldrich, UK).

**Figure 5—figure supplement 3.**
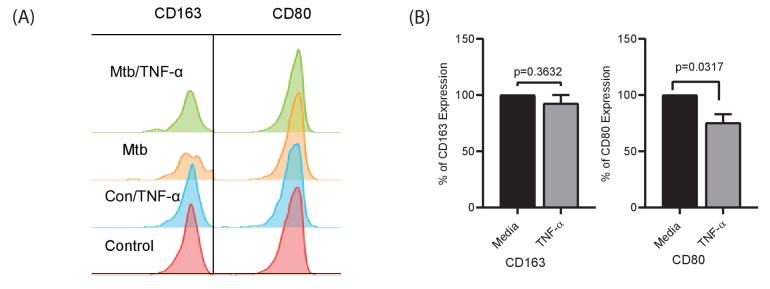
TNF-α skews polarization of monocytes to macrophages with lower CD80 expression. PBMCs were infected with Mtb H37Rv at MOI of 0.1 and encapsulated in alginate-collagen microspheres after overnight incubation. Microspheres were then incubated in complete RPMI (with L-Glutamine and 10% human serum) with TNF-α 7.5 ng/ml. Uninfected PBMCs were encapsulated and treated similarly as a comparator for TNF-α stimulation. At day 7, the microspheres were decapsulated in 0.5 mM EDTA solution at pH of 7.2. Double staining with CD14 and CD11b defined macrophages, which were classified by CD80 and CD163 expression. (**A**) Histogram showing expression of CD163 and CD80 where there was significant decrease in CD80 expression as shown if Figure (**B**). TNF suppressed the relative geometric mean of CD80, but did not affect CD163 expression. This experiment was performed in four separate donors.

**Figure 5—figure supplement 4.**
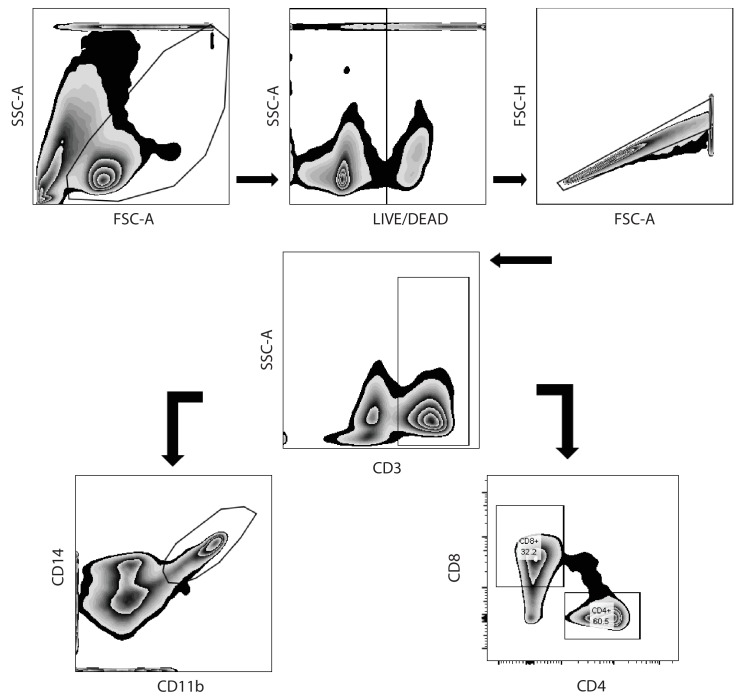
Hierarchical gating strategy used to identify lymphocyte and monocytic populations from decapsulated microspheres containing human peripheral blood monocular cells. Single cells were decapsulated from microspheres in 5 mM EDTA, washed and processed for flow cytometry. First doublets were excluded from live cells, then cells were gated as CD3+ and CD3-. Subsequently, lymphocytes were further classified into CD4+ and CD8+, which are sub-categorized based on PD-1 staining. Double staining with both CD14 and CD11b defined macrophages, which were further analysed for PD-L1, CD80 and CD163 surface expression. All the antibodies and clone number are listed in the text and the key resources table.

To explore the effect of TNF-α further, we then generated microspheres incorporating infected PBMC with and without anti-TNF-α neutralising antibodies. Consistent with our initial observation, anti-TNF-α neutralising antibodies suppressed the TNF-mediated increased Mtb growth, with a partial reduction at each concentration studied of two different neutralising antibodies (Figure 5E and Figure 5—figure supplement 2). Exogenous TNF-α modulated macrophage polarisation within microspheres, reducing CD80 expression at day 7 (Figure 5—figure supplement 3). Therefore, we next investigated whether anti-PD-1-induced Mtb growth could be reversed by blocking TNF-α activity. Anti-PD-1 antibody treatment of infected cells again augmented Mtb growth, and this increased growth could be reversed by the incorporation of anti-TNF-α antibodies into the microspheres (Figure 5F). This confirms that an excess of TNF-α is the primary driver of increased Mtb growth caused by PD-1 inhibition in the 3D model.

### TNF-α is highly expressed in TB granulomas and sputum TNF-α negatively correlates with circulating PD-1 expression

**Figure 6.**
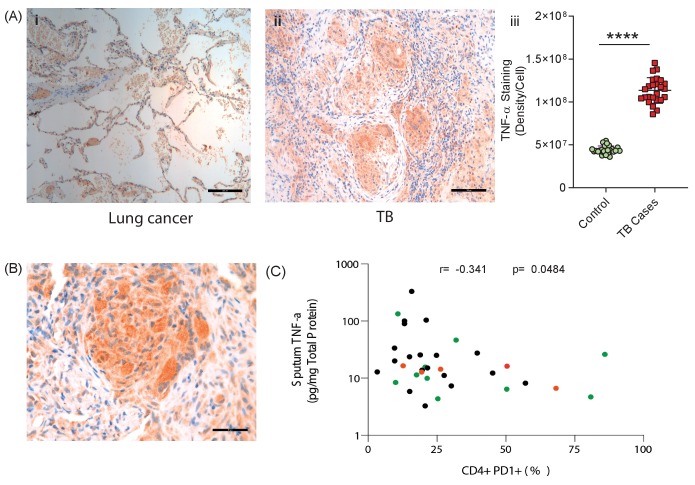
TNF-α is expressed in human TB granulomas and sputum TNF-α concentrations negatively correlate with circulating PD-1 expression. (**A**) TNF-α is expressed within human lung TB granulomas, with greater immunoreactivity than control lung tissue at the excision margin of lung cancer (i and ii). Quantification of TNF-α immunostaining (n = 5) in TB cases was significantly higher than controls (n = 5) (iii). (**B**) TNF-α immunostaining was extensive in the lung granuloma of a patient that developed TB whilst treated with pembrolizumab, a humanized anti-PD-1 antibody (n = 1). (**C**) CD4^+^ T cell PD-1 expression on circulating PBMC negatively correlates with sputum TNF-α concentration in a separate cohort where paired sputum and PBMCs samples are available. Green dots, healthy controls; Black TB cases; Orange respiratory symptomatics. Analysis by Spearman’s correlation analysis gave r-value of −0.341 with p=0.0484.

Finally, to establish the in vivo relevance of our cell culture model findings, we performed immunohistochemical analysis of biopsies from patients with standard TB and anti-PD-1 associated TB. TNF-α was expressed within TB granulomas, with greater immunoreactivity than control lung tissue at the excision margin of lung cancer (Figure 6A). Consistent with our cell culture observations, TNF-α immunostaining was extensive in a biopsy from a patient that developed pulmonary TB whilst treated with pembrolizumab, an anti-PD-1 antibody (Figure 6B). Quantitative analysis of differences between standard TB and anti-PD-1 TB was not possible due to the unique nature of this clinical specimen, as TB diagnosis is usually made by bronchoalveolar lavage as opposed to percutaneous biopsy.

We then analysed the association between PD-1 expression by circulating CD4^+^ and CD8^+^ T cells and sputum TNF-α concentrations in a previously reported cohort (Walker et al., 2017). Although this involved comparing PD-1 expression by circulating T cells, remote from the site of disease, with total TNF-α concentration in the sputum, we hypothesised that a reverse association would support the conclusion that PD-1 limits TNF-α secretion in patients. Consistent with this, CD4^+^ T cell PD-1 expression negatively correlated with sputum TNF-α, with a significant negative association despite the relatively low sample numbers, which would be expected to obscure an effect (Figure 6C).

## Discussion

Although Mtb kills more people than any other infection worldwide, an enduring enigma is that 90% of those exposed exert lifelong control of the pathogen. In seminal post-mortem studies when TB was highly prevalent in the United States, Opie showed that 30% of humans who died from other causes had viable Mtb present in the lung apices (Opie and Aronson, 1927). Therefore, in the majority of exposed individuals, a stable relationship forms between host immune cells and Mtb without disease developing. Disseminated TB disease often develops in immunocompromised individuals, such as advanced HIV infection, newborn infants or following anti-TNF-α therapy, and this observation informs the view that TB can arise as a disease of a deficient immune response to the pathogen (O'Garra et al., 2013).

However, the commonest form of human TB, and the one that leads to transmission, is apical pulmonary disease (Elkington et al., 2011). This occurs most frequently in young adults between the ages of 20–25 with the strongest recall response to Mtb antigens, as measured by the Mantoux test (Comstock et al., 1974). Therefore, these clinical observations demonstrate that infection results in a stable symbiosis between host and pathogen in the majority of individuals, and a pronounced immune response associates with the subsequent development of infectious pulmonary disease. The recently emerging clinical phenomenon of TB rapidly developing after initiating anti-PD-1 immunotherapy (Fujita et al., 2016; Lee et al., 2016; Chu et al., 2017; Picchi et al., 2018; Jensen et al., 2018; Elkington et al., 2018; He et al., 2018; Takata et al., 2019; Barber et al., 2019; Tsai et al., 2019; van Eeden et al., 2019) further reinforces that an excessive immune response in TB can be harmful.

PD-1 is expressed on T cells at the site of TB disease and PD-1 expression on circulating CD4+ T cells associates with bacterial load (Day et al., 1995). PD-1 expression is elevated in circulating CD4 T cells in TB (Shen et al., 2016) and has been proposed to limit an effective host immune response. Consequently PD-1 inhibition has been advanced as a therapeutic target to accelerate clearance of infection (Zumla et al., 2016; Shen et al., 2016; Jurado et al., 2008; Singh et al., 2013; Suarez et al., 2019; Jiang et al., 2014). However, from an evolutionary perspective, PD-1 is proposed to limit immunopathology in the face of chronic antigenic stimulation (Sharpe and Pauken, 2018). Therefore, it is equally plausible, and indeed perhaps more logical, that PD-1/PD-L1 pathway up-regulation in TB is a physiologically appropriate response to the persistent pathogen. We found that hypoxia further up-regulated the PD-1/PD-L1/2 axis, consistent with hypoxia increasing expression in cancer (Noman et al., 2014), and TB lesions are hypoxic both in model animals and human lesions (Belton et al., 2016; Via et al., 2008). Analysis of the effect of hypoxia is complicated that both host and pathogen physiology are altered, with hypoxia causing reduced Mtb growth (Devasundaram et al., 2016; Ortega et al., 2014; Eoh and Rhee, 2013) but also causing diverse host physiological changes. PD-1 may be particularly important in limiting excessive inflammation and pathology in conditions of low oxygen tension. TB reactivation following immune checkpoint blockade, and the extreme susceptibility of PD-1 deficient mice to Mtb infection (Lázár-Molnár et al., 2010; Barber et al., 2011), would support such a regulatory role, even though it runs counter to widely advanced disease paradigms.

Our work further highlights the double-edged sword of the host immune response in TB (Ehlers, 1999). TNF-α is clearly essential to an effective host immune response to TB, as disease frequently develops after treatment with anti-TNF-α antibodies (Keane et al., 2001), and TNF-α inhibits Mtb growth in zebrafish macrophages (Clay et al., 2008) and human alveolar macrophages (Hirsch et al., 1994). However, excessive TNF-α is also associated with poor outcomes in TB. In human cells, an excess of TNF-α can increase Mtb growth (Byrd, 1997; Engele et al., 2002), consistent with our observations. In patients with active TB, TNF-α expression by Mtb-specific T cells is increased (Harari et al., 2011; Tebruegge et al., 2015) and TNF-α associates with more severe radiological findings (Casarini et al., 1999; Tsao et al., 2000). In diverse model systems, excessive TNF-α has been shown to cause harmful inflammation (Bekker et al., 2000; Taylor et al., 2005; Tsenova et al., 1999; Roca and Ramakrishnan, 2013; Tzelepis et al., 2018), consistent with TNF-α exerting a bell-shaped effect on host immunity, with either deficit or excess detrimental. Similarly, we report a deleterious effect of additional GM-CSF, in contrast to GM-CSF improving control of Mtb growth in murine macrophages (Rothchild et al., 2014). This suggests that cytokine responses are highly dose and context dependent, potentially with each demonstrating non-linear responses leading to a complex matrix of what may denote the optimal cytokine profile for Mtb control. Consistent with our findings, in the one patient that developed TB on anti-PD-1 treatment where longitudinal samples are available, there was a spike in PPD-specific TNF-α expressing cells prior to with the development of active TB (Barber et al., 2019), consistent with PD-1 acting as a regulator of TNF-α expression in TB. Taken together, these findings demonstrate a harmful effect of excessive TNF-α in TB.

We found that multiple cytokines and chemokines were increased after PD-1 inhibition, and therefore events in humans are likely to be more complex than TNF-α excess alone. The augmented inflammation may have multiple harmful effects, such as recruitment of excessive inflammatory cells and destruction of the extracellular matrix (Cambier et al., 2017; Al Shammari et al., 2015; Mishra et al., 2017), which favour Mtb growth. In the mouse model of TB, one key role of PD-1 is to limit IFN-γ production (Sakai et al., 2016), and we have shown that excess IFN-γ also accelerates Mtb growth in microspheres (Tezera et al., 2017a). We did not show an effect of TNF-α on cell survival, although TNF-α has been reported to increase macrophage necrosis in the zebrafish model, via a mitochondrial-lysosomal-endoplasmic reticulum circuit (Roca and Ramakrishnan, 2013; Roca et al., 2019). The effect of PD-1 inhibition on macrophage polarisation and survival warrants further investigation, as this is likely to be one determinant of outcome (Lerner et al., 2017; Cronan et al., 2016).

As in all in vitro systems, the bioelectrospray model has limitations (Elkington et al., 2019). For example, it does not permit the ingress of new inflammatory cells, and therefore can only be used to investigate the early events resulting from anti-PD-1 treatment, and not the recruitment of inflammatory cells by increased chemokine secretion. In addition, although it permits longer analysis of the host-pathogen interaction than other human primary cell culture systems, the 3 week standard experiment remains shorter than human infection, and so it models early events. We have not yet characterised which cells produce TNF-α, nor the wider phenotypic changes that result from inhibition of the PD-1 axis. The optimal approach would be to integrate single cell RNAseq to the analysis pipeline, so the phenotype of different cells can be comprehensively analysed. As microspheres can be readily dissolved by incubation in EDTA to release the cells, the system is suited for this technical development.

The common side effects of anti-PD-1 treatment in patients are termed immune related Adverse Events (irAEs), which are autoimmune in nature (Postow et al., 2018). We have proposed that an autoimmune process may exacerbate pathology in TB (Elkington et al., 2016), and others have suggested that a loss of tolerance underlies progression to active TB (Divangahi et al., 2018; Olive and Sassetti, 2018). The common theme is that an excessive response to antigens, whether host or pathogen-derived, can drive disease in TB and our findings further support this conclusion. PD-1 may act to fine tune the balance between pro- and anti-inflammatory responses necessary to control infection without causing pathology. Notably, immune related adverse events to immune checkpoint inhibitors may be treated with anti-TNF-α antibodies (Postow et al., 2018), suggesting TNF-α may be the primary driver of both autoimmunity and TB pathology after PD-1 treatment.

Our model provides the mechanistic insights into a clinical phenomenon with significant implications for future TB treatment and vaccine approaches. Simply driving a stronger immune response to Mtb seems unlikely to be beneficial, as clinical and epidemiological data suggest it may be harmful. For example, host-directed therapies may be designed to accelerate bacterial clearance whilst concurrently reducing immunopathological effects by appropriate skewing of macrophage phenotype. A more nuanced view considering the different immunological phases of TB is essential, differentiating events at the point of initial exposure from the late events at the apex of the lung, where excessive inflammation leads to immunopathology and transmission (Elkington and Friedland, 2015). Defining the distinction between a protective and pathological immune response in human TB remains a key unanswered question, essential to inform new interventions to control the TB pandemic.

## Materials and methods

**Key resources table:** 

Reagent type (species) or resource	Designation	Source or reference	Identifiers	Additional information
Strain, strain background (*Mycobacterium tuberculosis*)	H37Rv	(From Ref: 20)		Used at multiple of infection of 0.1
Strain, strain background (*Mycobacterium tuberculosis*)	H37Rv pMV306hsp+LuxAB+G13+CDE	(From Ref: 20)		Used at multiple of infection of 0.1
Strain, strain background (*Mycobacterium tuberculosis*)	H37Rv pMV306hsp encoding the wild-type FFluc	(From Ref: 20)		Used at multiple of infection of 0.1
Biological sample (Antibodies)				
Antibody	anti-CD45-V500 Horizon (Clone no. HI30)	BD Biosciences	Cat.No.563792	Monoclonal mouse antibody
Antibody	anti-CD3 Brilliant Violet 785 (Clone no. OKT3)	Biolegend	Cat.No.317330	Monoclonal mouse antibody
Antibody	anti-CD3-PE (Clone no. HIT3a)	Biolegend	Cat.No.300308	Monoclonal mouse antibody
Antibody	anti-CD4 Brilliant Ultra Violet 496 (Clone no. SK3)	BD Bioscience	Cat.No.612937	Monoclonal mouse antibody
Antibody	anti-CD4-PerCP (Clone no. OKT4)	Biolegend	Cat.No.317432	Monoclonal mouse antibody
Antibody	anti-CD8 Brilliant Violet 605 (Clone no. RPA-T8)	Biolegend	Cat.No.301040	Monoclonal mouse antibody
Antibody	anti-CD8-APC (Clone no. SK1)	Biolegend	Cat.No.344722	Monoclonal mouse antibody
Antibody	anti-CD103-APC (Clone no. Ber-ACT8)	BD Biosciences	Cat.No.563883	Monoclonal mouse antibody
Antibody	anti-CD69 Brilliant Ultra Violet 395 (Clone no. FN50)	Biolegend	Cat.No.310902	Monoclonal mouse antibody
Antibody	anti-HLA-DR-PerCP (Clone no. L243)	Biolegend	Cat.No.307628	Monoclonal mouse antibody
Antibody	anti-CD279-BB515 (Clone no. EH12.1)	BD Biosciences	Cat.No.564494	Monoclonal mouse antibody
Antibody	anti- CD-274-BB515 (Clone no. MIH1)	BD Biosciences	Cat.No.564554	Monoclonal mouse antibody
Antibody	anti-PD-1 Brilliant Violet 421 (Clone no.EH12.1)	BD Biosciences	Cat.No.562516	Monoclonal mouse antibody
Antibody	anti-CD11b-APC (Clone no. ICRF44)	Biolegend	Cat.No.301310	Monoclonal mouse antibody
Antibody	anti-CD45-APC/Cy7 (Clone no. 2D1)	Biolegend	Cat.No.368516	Monoclonal mouse antibody
Antibody	anti-CD14-AP/APC (Clone no. HCD14)	Biolegend	Cat.No.325608	Monoclonal mouse antibody
Antibody	anti-True-Stain Monocyte Blocker	Biolegend	Cat.No.426102	Monoclonal mouse antibody
Antibody	anti-CD4 (Clone no. M7310)	DAKO	Cat.No.M7310	Monoclonal mouse antibody
Antibody	anti-CD8 (Clone no. M7103)	DAKO	Cat.No. M7103	Monoclonal mouse antibody
Antibody	anti-PD1 (Clone no. ab5287)	Abcam	Cat.No.ab52587	Monoclonal mouse antibody
Antibody	anti-TNF-α (Clone no. ab1793)	Abcam	Cat.No.ab1793	Monoclonal mouse antibody
Antibody	Spartalizumab	Selleckchem	Cat.No.A2017	20 µg/ml and 200 µg/ml, monoclonal, mouse IgG4
Antibody	IgG4	Sino Biologicals	Cat.No.13505-HNAH	20 µg/ml and 200 µg/ml,monoclonal, mouse IgG4
Antibody	Mouse IgG1 kappa Isotype Control (P3.6.2.8.1),	Thermo Fisher Scientific	Cat.No.16-4714-82	50 µg/ml, mouse monoclonal IgG2A
Antibody	Mouse IgG1 Negative Control, clone Ci4	Merck Life Sciences	Cat.No.MABC002	51 µg/ml, mouse monoclonal IgG2A
Antibody	anti-TNF-α	Thermo Fisher Scientific	Cat.No.16-7348-81	52 µg/ml, mouse monoclonal IgG2A
Sequence-based reagent (Applied Biosystems TaqMan Gene Expression primers)	GAPDH	Thermo Fisher Scientific	#Hs02758991_g1	
	β2-Microbulin	Thermo Fisher Scientific	#Hs00608023_m1	
	FNTA	Thermo Fisher Scientific	#Hs00357739_m1	
	PDCD1	Thermo Fisher Scientific	#Hs01550088_m1	
	CD274	Thermo Fisher Scientific	#Hs00204257_m1	
	PDCD1LG2	Thermo Fisher Scientific	#Hs00228839_m1	
	HIF-1α	Thermo Fisher Scientific	#Hs00153153_m1	
Commercial assay or kit	CytoTox-Glo Cytotoxicity Assay	Promega	G9291	Commercial assay or kit
	Lactate Dehydrogenase Activity Assay Kit	Merck	11 644 793 001	Commercial assay or kit
	Cytokine and Chemokine 35-Plex Human ProcartaPlex Panel	Thermo Fisher Scientific	LHC6005M	Commercial assay or kit
Chemical compound, drug	PD-1/PD-L1 Inhibitor 1	Cambridge Biosciences, UK	#1675201-83-8	Chemical compound
	Recombinant Human G-CSF	ImmunoTools	Cat.No.11343115	1 ng/ml and 5 ng/ml
	Recombinant Human GM-CSF	ImmunoTools	Cat.No.11343125	0.25 ng/ml and 1.25 ng/ml
	Recombinant Human IL-1RA/IL1 F3	ImmunoTools	Cat.No.11344876	1.25 ng/ml and 6.25 ng/ml
	Recombinant Human IL-10	ImmunoTools	Cat.No.11340105	0.2 ng/ml and 1 ng/ml
	Recombinant Human IL-6	ImmunoTools	Cat.No.11340066	10.0 ng/ml and 50.0 ng/ml
	Recombinant Human IL-12	ImmunoTools	Cat.No.11349125	0.5 ng/ml and 2.5 ng/ml
	Recombinant Human TNF-α	ImmunoTools	Cat.No.11343017	0.3 ng/ml, 1.5 ng/ml,7.5 ng/ml and 15 ng/ml
	Recombinant Human IL-15	ImmunoTools	Cat.No.11340155	0.5 ng/ml, 5 ng/ml and 50 ng/ml
	Recombinant Human IL-17A	ImmunoTools	Cat.No.11340176	1 ng/ml, 10 ng/ml and 100 ng/ml
	Recombinant Human IL-17F	ImmunoTools	Cat.No.11349176	1 ng/ml, 10 ng/ml and 100 ng/ml
	Recombinant Human RANTES	ImmunoTools	Cat.No.11343196	0.3 ng/ml and 1.5 ng/ml
	Recombinant Human MIP-1α	ImmunoTools	Cat.No.11343206	1.5 ng/ml and 7.5 ng/ml
	Recombinant Human MIP-1β	ImmunoTools	Cat.No.11343223	1.0 ng/ml and 5 ng/ml
	Recombinant Human MCP	ImmunoTools	Cat.No.11343386	1 ng/ml, 10 ng/ml and 100 ng/ml
Software, algorithm	FlowJo	BD Bioscences	version 10.6.1	Software
	BD FACSDiva Software	BD Biosciences		Software
	Graphpad Prism	GraphPad Software LLC	v7.05	Software

### *M. tuberculosis* culture

*M. tuberculosis H37Rv* (Mtb) was cultured in Middlebrook 7H9 medium (supplemented with 10% ADC, 0.2% glycerol and 0.02% Tween 80) (BD Biosciences, Oxford). Bioluminescent Mtb containing luxABCDE (Mtb lux+) and Mtb expressing ffLuc (Mtb ffLuc+) were cultured with kanamycin 25 μg/ml. Luminescence was measured with either GloMax 20/20 single tube luminometer (Promega,UK) or GloMax Discover microplate reader (Promega,UK). Cultures at 1 × 10^8^ CFU/ml Mtb (OD = 0.6) were used for all experiments at multiplicity of infection (MOI) of 0.1. Live Mtb was used in all experiments apart from the time lapse microscopy, which used UV killed TB. Mtb colony counting was performed by serial dilution on Middlebrook 7H11 Agar. Bioluminescence from the Mtb ffLuc+ was induced using D-luciferin (ThermoFisher, UK) at a concentration of 750 μM in Hank’s balanced salt solution (HBSS).

### PBMC cell isolation from human blood

For the 3D microsphere experiments, PBMC were separated from single donor leukocyte cones (National Health Service Blood and Transfusion, Southampton, UK) by density gradient centrifugation over Ficoll-Paque (GE Healthcare Life Sciences). Ethical approval for these studies was provided by the National Research Ethics Service Committee South Central - Southampton A, ref 13/SC/0043.

Study participants for the correlation analysis of sputum-TNF-α with PD-1 expression on CD4^+^ and CD8^+^ T cells are the cross-sectional study individuals in a previously reported cohort (Walker et al., 2017; Walker et al., 2019). Participants were recruited from an outpatient clinic in Khayelitsha, South Africa and were either healthy volunteers, non-TB respiratory symptomatics or recently diagnosed TB patients. Flow cytometric analysis was performed on cryopreserved PBMC isolated from whole blood as previously reported (Walker et al., 2019). The study was approved by the University of Cape Town Human Research Ethics Committee (REF 516/2011) and conducted in accordance with the Declaration of Helsinki.

### Analysis of PD-1 expression in blood and lung of TB patients

Lung tissue and matched PBMC were obtained from the AHRI Lung study cohort approved by the Biomedical Research Ethics Committee (BREC) of the University of Kwa-Zulu Natal, BREC reference: BE019/13. All participants underwent surgical resection to treat TB related lung complications, including haemoptysis, bronchiectasis, persistent cavitatory disease, shrunken or collapsed lung or drug-resistant infection, at the King Dinuzulu Hospital in Durban, KwaZulu- Natal and Inkosi Albert Luthuli Central Hospital (IALCH) in Durban, KwaZulu-Natal. PBMC were isolated from whole blood using standard Ficoll-Histopaque (Sigma) density gradient centrifugation by standard protocol.

Lung tissues was cut into approximately 1 mm^3^ pieces, washed several times with cold HBSS (Lonza) and re-suspended in 8mls of pre-warmed digestion media R10 (RPMI supplemented with 10% FCS, 2 mM L-glutamate, 100 U/ml Penstrep), containing 0.5 mg/ml collagenase D (Roche) and 40 U/ml DNaseI (Roche), and transferred to GentleMACS C-tubes (Miltenyi) for mechanical digestion according to the manufacturer’s instructions. The suspension was incubated for 30 min at 37°C, followed by an additional mechanical digestion step and another 30 min incubation step at 37°C. The final suspension was passed through a 70 μm cell strainer and washed twice in HBSS. PBMC and lung cells were phenotyped by surface staining with a near-infrared live/dead cell viability cell staining kit (Invitrogen) and a cocktail of fluorochrome conjugated antibodies: αCD45-V500 Horizon clone HI30 (BD Biosciences), αCD3 Brilliant Violet 785 clone OKT3 (Biolegend), αCD4 Brilliant Ultra Violet 496 clone SK3 (BD Bioscience), αCD8 Brilliant Violet 605 clone RPA-T8 (Biolegend), αCD103-APC clone Ber-ACT8 (BD Biosciences), αCD69 Brilliant Ultra Violet 395 clone FN50 (Brilliant Horizon), αPD-1 Brilliant Violet 421 clone EH12.1 (BD Biosciences). Cells were stained with 25 uL of antibody cocktail in the dark for 20 min at room temperature followed by washing with PBS, then fixed in 2% PFA. Data was acquired using BD Aria Fusion cytometer and analyzed using FlowJo Software v.9.9 (Treestar Inc, Ashland, OR).

### Immunohistochemistry of paraffin-fixed tissue

Immunohistochemical analysis was performed on paraffin-embedded lung tissue from patients with pulmonary TB and lung cancer that were mounted at 4 µm thin onto APS coated glass slides and dried, using the following antibodies: anti-CD4 (Clone no. M7310) (DAKO), anti-CD8 (Clone no. M7103) (DAKO), anti-PD1 (Clone no. ab5287) (Abcam) and anti-TNF-α (Clone no. ab1793) (Abcam). Staining was done at optimised concentrations usingrecommended buffers for each antibody.

### Microencapsulation of cells

Microspheres were generated with an electrostatic generator (Nisco, Zurich, Switzerland) as described previously (Tezera et al., 2017b). Briefly, PBMC were infected overnight with Mtb in a 250 cm^2^ flask, cells were detached, pelleted and mixed with 1.5% sterile alginate (Pronova UP MVG alginate, Nova Matrix, Norway) and 1 mg/mL collagen (Advanced BioMatrix, USA) at a final concentration of 5 × 10^6^ cells/ml. The cell-alginate suspension was injected into the bead generator where microspheres were formed in an ionotropic gelling bath of 100 mM CaCl_2_ in HBSS. After washing twice with HBSS with Ca^2+^/Mg^2+^, microspheres were transferred in RPMI 1640 medium containing 10% human AB serum and incubated at 37°C. Microspheres were either dispensed into eppendorfs, which were then randomly allocated to different environmental conditions, or plated into a 96-well plate with conditions in triplicate according to a pre-determined template. For experiments in hypoxia, microspheres were incubated in 1% oxygen in Galaxy 48 R CO_2_ incubator (Eppendorf, UK) until analysis. Supernatants were collected at defined time points. Time points described are days post infection.

### Live cell imaging

Uninfected or UV killed Mtb infected cells suspended in alginate-collagen matrix were plated in an eight well μ-Slide (ibidi GmbH, Germany). HBSS containing Ca^2+^ was used for cross-linking of alginate in the extracellular matrix for 15 min and then replaced with RPMI medium with 10% human AB serum. Samples were imaged using an Olympus IX81 time-lapse microscope with temperature of 37°C and CO_2_ concentration 5%. Z-stacks 200 µm in height were captured at one position in each sample every 30 min for 48 hr. Images were exported as tif files and opened in ImageJ.

### PD-1/PD-L1 Inhibition

Small chemical inhibition of PD-1/PD-L1 signalling was by PD-1/PD-L1 Inhibitor 1 (CAS Registry #1675201-83-8, Cambridge Biosciences, UK), a compound that competitively blocks the interaction of PD-1 with it ligand protein PD-L1 (Guzik et al., 2017). Inhibitor one was prepared in DMSO (Sigma-Aldrich,UK) at a concentration of 6.3 mM and dissolved to a concentration of 1 nM, 10 nM, 100 nM and 1 μM in complete media and added to media around microspheres on the day after encapsulation. Luminescence was monitored on specific days. For the microspheres incubated in hypoxic conditions, measurement of luminescence was repeated for every 30 min after the addition of luciferin until the reading plateaued, which was usually 2 hr.

In antibody inhibitory experiments, spartalizumab (Selleckchem, Germany), a humanised IgG4 anti-PD1 monoclonal antibody, was used to inhibit of PD-1/PD-L1 signalling in microspheres. Briefly, cells were infected with Mtb overnight, pelleted and then a suspension of anti-PD1 antibodies (20 and 200 μg/ml) were added and pre-incubated for 1 hr. Cells were then encapsulated within microspheres and kept for 14 days in either normoxia or 1% oxygen at 37°C and 5% CO_2_. Mtb growth was measured using luminescence and supernatants were taken for either cytokine or LDH measurement. An IgG4 human antibody was used at the same concentration as a control.

### Cell toxicity assays

Lactate dehydrogenase (LDH) release in the supernatants collected at different time points was analysed by a colorimetric activity assay as per manufacturer’s instructions (Roche, Burgess Hill, United Kingdom). As a second assay, CytoTox-Glo Cytotoxicity Assay (Promega) was used, which measures dead-cell protease activity released from cells without membrane integrity using a luminogenic peptide substrate, the AAF-Glo Substrate. Luminescence from 96-well plates was analysed by GloMax Discover (Promega). The LDH assay was suited for later time points, as this could be performed on microspheres in eppendorfs, while the CytoTox glow required analysis in 96 well plates and so was best suited to analysis in the first week.

### Gene expression analysis

All the reagents were sourced from ThermoFisher Scientific (Paisley, UK). In brief, microspheres were decapsulated with 5 mM EDTA, cells were pelleted and immediately lysed using TRIzol Reagent. RNA was transcribed using High Capacity cDNA Reverse Transcription kit. TaqMan Universal master mix and primers specific for genes were GAPDH (#Hs02758991_g1), β2-Microbulin (#Hs00608023_m1), FNTA (#Hs00357739_m1), PDCD1 (#Hs01550088_m1), CD274 (#Hs00204257_m1), PDCD1LG2 (#Hs00228839_m1) and HIF-1α (Hs00153153_m1) were used for qPCR according to the manufacturer’s instructions and the comparative threshold (CT) method was employed to analyse all qPCR data.

### Immunophenotyping of cells from microspheres

Microspheres were decapsulated with 5 mM EDTA in HBSS with no Ca^2+^/Mg^2+^ at day seven after encapsulation and 2 million cells prepared for staining in RPMI with 5% foetal bovine serum. To measure the expression of PD-1 in CD4^+^ and CD8^+^ T cells and PD-L1 expression in CD14^+^CD11b^+^ cells, the following antibody panel was used: CD3-PE (clone HIT3a, Biolegend), HLA-DR-PerCP (clone L243, Biolegend), CD4-PerCP (clone OKT4, Biolegend), CD8-APC (clone SK1, Biolegend), CD11b-APC (clone ICRF44, Biolegend), CD45-APC/Cy7 (clone 2D1, Biolegend), CD14-AP/APC (clone HCD14, Biolegend), CD279-BB515 (Clone EH12.1, BD), CD-274-BB515 (Clone MIH1, BD) and True-Stain Monocyte Blocker (Biolegend, USA). Gates were defined using fluorescence minus one control after exclusion of the dead cells using Live/Dead fixable stain (ThermoFisher, UK). Gating strategy is provided in Figure 5—figure supplement 4. Cells were acquired after fixing them in 2% paraformaldehyde in HBSS for 1 hr using FACSAria (Becton Dickinson, UK) and analysed by FACSDiva software (Becton Dickinson) and Flow Jo version 10 (Treestar).

### Cytokine supplementation

Microspheres were incubated in RPMI 1640 with 10% AB serum in an opaque 12-well tissue culture plate with G-CSF, GM-CSF, IL-1RA, IL-6, IL-10, IL-12, IL-15, IL-17A, IL-17F, TNF-α, RANTES, MIP-1α, MIP-1β and MCP at two concentrations determined by the cellular experiments, at 37°C and 5% CO_2_. All the cytokines were purchased from ImmunoTools (Germany), suspended in RPMI with 0.1% human serum and kept at −80°C until use. Bacterial growth was monitored with luminescence using GloMax Discover microplate reader (Promega,UK).

### Luminex analysis

Samples were sterilised by filtration through a 0.22 μM Durapore membrane (MerkMillipore). Concentrations of cytokines (ThermoFisher, UK) were determined using a Bioplex 200 platform (Bio-Rad, UK) according to the manufacturer’s protocol and quantified per milligram of total protein measured by Bradford assay (Biorad).

### Statistical analysis

All experiments were performed on a minimum of 2 occasions from separate donors as biological replicates and on each occasion with a minimum of 3 technical replicates. Some donor-to-donor variation occurred in terms of absolute RLU, as expected in the analysis of primary human cells, but the direction of effects were always consistent. Data presented are from a representative donor and include the mean and SEM. Analysis was performed in Graphpad Prism v7.05. Students t-test was used to compare pairs and ANOVA with Tukey’s correction for multiple comparisons for groups of 3 or more groups where it was appropriate. For the flow cytometric analysis of clinical samples, data were analysed using Mann-Whitney test for comparing pairs and Kruskal-Wallis test with Dunn’s multiple comparisons test for three or more group.

## Data Availability

All data generated or analysed during this study are included in the manuscript and supporting files. Source data files have been provided for for all figures as a data resource file.

## Additional files

Source data 1. Primary data for figures provided in manuscript.

Supplementary file 1. PD-1 expressing cells for each subset expressed as percentage of live CD45+ cells, with range in parentheses.

Transparent reporting form
