# Supplementary material for: Anti-PD-1 immunotherapy leads to tuberculosis reactivation via dysregulation of TNF-α
Source: eLife. 2020 Feb 24;9:e52668. doi: 10.7554/eLife.52668 (PMC7058383; doi:10.7554/eLife.52668)
Supplement: Transparent reporting form [file elife-52668-transrepform.docx]

***eLife’s* transparent reporting form**

We encourage authors to provide detailed information *within their submission* to facilitate the interpretation and replication of experiments. Authors can upload supporting documentation to indicate the use of appropriate reporting guidelines for health-related research (see [EQUATOR Network](http://www.equator-network.org/%20)), life science research (see the [BioSharing Information Resource](https://biosharing.org/)), or the [ARRIVE guidelines](http://www.plosbiology.org/article/info:doi/10.1371/journal.pbio.1000412) for reporting work involving animal research. Where applicable, authors should refer to any relevant reporting standards documents in this form.

If you have any questions, please consult our Journal Policies and/or contact us: [editorial@elifesciences.org](mailto:editorial@elifesciences.org).

**Sample-size estimation**

- You should state whether an appropriate sample size was computed when the study was being designed
- You should state the statistical method of sample size computation and any required assumptions
- If no explicit power analysis was used, you should describe how you decided what sample (replicate) size (number) to use

Please outline where this information can be found within the submission (e.g., sections or figure legends), or explain why this information doesn’t apply to your submission:

The sample size for the clinical study was based on previous similar studies, as no preliminary data were available to inform a sample size calculation. Where previously acquired and reported datasets were re-analysed, all samples available for analysis were studied, (Walker et al Clin Inf Dis 2017 65:121, Walker et al Clin Inf Dis 2019, ePub ahead of press).

The immunohistochemistry is non-quantitative and therefore a sample size calculation is not possible.

**Replicates**

- You should report how often each experiment was performed
- You should include a definition of biological versus technical replication
- The data obtained should be provided and sufficient information should be provided to indicate the number of independent biological and/or technical replicates
- If you encountered any outliers, you should describe how these were handled
- Criteria for exclusion/inclusion of data should be clearly stated
- High-throughput sequence data should be uploaded before submission, with a private link for reviewers provided (these are available from both GEO and ArrayExpress)

Please outline where this information can be found within the submission (e.g., sections or figure legends), or explain why this information doesn’t apply to your submission:

All microsphere experiments were performed on a minimum of two occasions on different dates and studying different donors, with a minimum of three technical replicates for every condition per donor. These details are provided in the statistical analysis section of the methods. No outliers were excluded. No high throughput sequence data were generated.

**Statistical reporting**

- Statistical analysis methods should be described and justified
- Raw data should be presented in figures whenever informative to do so (typically when N per group is less than 10)
- For each experiment, you should identify the statistical tests used, exact values of N, definitions of center, methods of multiple test correction, and dispersion and precision measures (e.g., mean, median, SD, SEM, confidence intervals; and, for the major substantive results, a measure of effect size (e.g., Pearson's r, Cohen's d)
- Report exact p-values wherever possible alongside the summary statistics and 95% confidence intervals. These should be reported for all key questions and not only when the p-value is less than 0.05.

Please outline where this information can be found within the submission (e.g., sections or figure legends), or explain why this information doesn’t apply to your submission:

Data presented are from a representative donor and include the mean and SEM. Analysis was performed in Graphpad Prism v7.05. Students t-test was used to compare pairs and ANOVA with Tukey’s correction for multiple comparisons for groups of 3 or more groups where it was appropriate. For the flow cytometric analysis of clinical samples, data were analysed using Mann-Whitney test for comparing pairs and Kruskal-Wallis test with Dunn’s multiple comparisons test for 3 or more group.

The statistical analyses are stated in the methods and the figure legends.

(For large datasets, or papers with a very large number of statistical tests, you may upload a single table file with tests, Ns, etc., with reference to sections in the manuscript.)

**Group allocation**

- Indicate how samples were allocated into experimental groups (in the case of clinical studies, please specify allocation to treatment method); if randomization was used, please also state if restricted randomization was applied
- Indicate if masking was used during group allocation, data collection and/or data analysis

Please outline where this information can be found within the submission (e.g., sections or figure legends), or explain why this information doesn’t apply to your submission:

After generation, microspheres were dispensed into either eppendorfs or 96 well plates. The eppendorfs were randomly allocated to the different intervention groups, whilst the plate layout for interventions was as predetermined by the experimental plan. This information is included in the methods section on page 17.

**Additional data files (“source data”)**

- We encourage you to upload relevant additional data files, such as numerical data that are represented as a graph in a figure, or as a summary table
- Where provided, these should be in the most useful format, and they can be uploaded as “Source data” files linked to a main figure or table
- Include model definition files including the full list of parameters used
- Include code used for data analysis (e.g., R, MatLab)
- Avoid stating that data files are “available upon request”

Please indicate the figures or tables for which source data files have been provided:

All source data will be made available via the University of Southampton ePrints research repository with a key linking to each main and supplementary figure in the manuscript
